# Role of Cross-Cleft Contacts in NMDA Receptor Gating

**DOI:** 10.1371/journal.pone.0080953

**Published:** 2013-11-21

**Authors:** Meaghan A. Paganelli, Cassandra L. Kussius, Gabriela K. Popescu

**Affiliations:** 1 Neuroscience Program, University at Buffalo, Buffalo, New York, United States of America; 2 Department of Biochemistry, University at Buffalo, Buffalo, New York, United States of America; University of Houston, United States of America

## Abstract

In response to brief glutamate exposure, NMDA receptors produce excitatory currents that have sub-maximal amplitudes and characteristically slow kinetics. The activation sequence starts when glutamate binds to residues located on the upper lobe of extracellularly located ligand-binding domains (LBDs) and then contacts lower lobe residues to bridge the cleft between the two hinged lobes. This event stabilizes a narrow-cleft LBD conformation and may facilitate subsequent inter-lobe contacts that further stabilize the closed cleft. Agonist efficacy has been traced to the degree of agonist-induced cleft-closure and may also depend on the stability of the closed-cleft conformation. To investigate how cross-cleft contacts contribute to the amplitude and kinetics of NMDA receptor response, we examined the activation reaction of GluN1/GluN2A receptors that had single-residue substitutions at the interface between LBD lobes. We found that side-chain truncations at residues of putative contact between lobes increased glutamate efficacy through independent additive mechanisms in GluN1 and GluN2A subunits. In contrast, removing side-chain charge with isosteric substitutions at the same sites decreased glutamate efficacy. These results support the view that in GluN1/GluN2A receptors’ natural interactions between residues on opposing sides of the ligand-binding cleft encode the stability of the glutamate-bound closed-cleft conformations and limit the degree of cleft closure, thus contributing to the sub-maximal response and emblematically slow NMDA receptor deactivation after brief stimulation.

## Introduction

Glutamate-activated channels sensitive to the synthetic agonists AMPA, kainate, or NMDA mediate almost all fast excitatory transmission between central neurons. Among these, NMDA receptors have several characteristic features that make them uniquely suited to the functions they serve in synaptic physiology: high calcium permeability, voltage-dependent block by magnesium ions, and distinctively slow deactivation kinetics (reviewed in 1). Relative to AMPA and kainate receptors, NMDA receptors activate and deactivate 10 to 100-fold slower and only a minority (< 30%) of the total number of glutamate-occupied channels contributes to the peak current response ([2–4], reviewed in [5]). The structural origins of the NMDA receptor slow kinetics and sub-maximal peak response are unknown.

Ionotropic glutamate receptors are tetramers of homologous subunits and share some aspects of their activation mechanisms [6,7]. The activation reaction is initiated by direct binding of neurotransmitter molecules to extracellular ligand-binding domains (LBDs), specifically within a cleft formed by two hinged lobes, D1 and D2 [8–13]. When genetically excised from functional receptors, LBDs form soluble proteins that maintain native-like pharmacology [14]. High-resolution structural data for a large number of ligand-LBD complexes have established that agonists form multidentate contacts with residues located on the two opposing lobes and subsequently facilitate direct cross-cleft interactions between D1 and D2 residues [15]. Together these interactions help to stabilize a subset of closed-cleft conformations that is characteristic for each ligand-LBD complex [16,17]. 

The degree of cleft closure varies across the solved ligand-bound LBD structures according to both the nature of the bound ligand and the LBD protein [11–13,18]. In addition, the magnitude of the receptor response to a given agonist correlates with the degree of ligand-induced cleft-closure and with the stability of closed-cleft conformations [19–21]. These findings led to the widely held hypothesis that the binding event promotes narrow-cleft conformations and facilitates the formation of direct inter-lobe contacts, which help keep the clefts closed longer. To analyze how cross-cleft D1-D2 contacts contribute to NMDA receptor activation, we examined the gating kinetics of receptors with point mutations in the LBD of GluN1 (N1) and GluN2A (N2A) subunits. Results showed that side-chain truncations increased gating whereas simply neutralizing charge decreased gating. Based on these results, we propose that specific interactions across the LBD clefts of N1 and N2A subunits control the degree of cleft closure and/or the stability of the closed-cleft conformation, and each of these effects have distinct contributions to the observed glutamate-elicited NMDA receptor response.

## Materials and Methods

### Cell culture and protein expression

HEK293 cells were maintained in Dulbecco’s Modified Eagle Medium (DMEM, Invitrogen) with 10% Fetal Bovine Serum at 37°C in 5% CO_2_. Cells were plated in 35 mm dishes for 24 hours before transfections. 

Plasmids encoding rat GluN1 (UO8261) and GluN2A (M91561) were subcloned into pcDNA3.1. Residues were selected for substitution based on predictions from the glycine-bound (PDB: 1PB7) and glutamate-bound (PDB: 2A5S) GluN1-LBD crystal structures. The following single-residue substitutions were introduced: K483A and K483M in GluN1, and K487A and N687L in GluN2A, where numbering included the signal peptide. 

Plasmids were transfected using the calcium phosphate method at a ratio N1:N2A:GFP = 1:1:1. After 2 hours, the transfection medium was washed and cells were changed into DMEM supplemented with 2 mM Mg^2+^ to prevent glutamate toxicity. Cells were used for electrophysiological recordings 24 - 48 hours post transfection.

### Electrophysiology

Single-channel currents were recorded using the cell-attached patch-clamp technique as described in detail previously [22]. Fire polished pipettes (12 - 25 MΩ) were filled with solutions containing (in mM): 1 glutamate, 0.1 glycine, 150 NaCl, 2.5 KCl, 10 HEPBS, 1 EDTA at pH 8.0 (NaOH). Inward sodium currents were recorded with QuB software (www.qub.buffalo.edu); were amplified, low pass filtered at 10 kHz, at an applied pipette potential of +100 mV with Axopatch 200B (Molecular Devices); and were sampled at 40 kHz (NIDAQ board) into digital files. 

Macroscopic currents were recorded with the whole-cell patch-clamp technique. Fire polished pipettes (4 - 6 MΩ) were filled with an intracellular solution containing (in mM): 135 CsF, 33 CsOH, 2 MgCl_2_, 1 CaCl_2_, 10 HEPES, and 11 EGTA at pH 7.4 (CsOH). Extracellular solutions, which contained (in mM): 1 glutamate, 0.1 glycine, 150 NaCl, 2.5 KCl, 0.01 EDTA, 10 HEPBS, 0.5 CaCl_2_ at pH 8.0 (NaOH), were superfused onto the recorded cell to elicit currents while clamping the cell at -70 mV. Currents were amplified, filtered at 2 kHz (Axopatch 200B), sampled at 5 kHz (Digidata 1440A) and acquired into digital files using pClamp suite software (Axon Instruments). 

Responses from excised patches were recorded from outside-out membrane patches using the same solutions as described above for whole-cell experiments. Two extracellular solutions, without (wash) or with 1 mM glutamate, were applied simultaneously through at 2.0 mm diameter glass theta tube (Harvard Apparatus) mounted on a piezoelectric translator (Burleigh LSS-3100/3200) to create a fast exchange between the two solutions onto the patch pipette. Open-tip potentials were measured at the end of each experiment to confirm that the 10-90% exchange occurred faster than 0.25 ms. Recorded currents were low-pass filtered at 5 kHz (Axopatch 200B), sampled at 50 kHz (Digidata 1440A) and stored as digital files with pClamp software.

### Kinetic analyses

Single-channel currents were converted from an analog signal to a digital file using a National Instruments A/D card and QuB acquisition software. Channel openings are denoted by downward deflections of 7 - 10 pA, whereas channel closures are designated by the absence of recorded current (0 pA). Segmental k-means (SKM) algorithm based on hidden Markov model was used to idealize the single channel data [23]. Each data point within the record was assigned to a conductance class (open or closed) based on the SKM algorithm. Subsequently, all the kinetic modeling was done using the idealized data after imposing a dead-time of 0.15 ms. A standard 5C4O model was used to fit state models directly onto the idealized data using a maximum interval log-likelihood (MIL) algorithm. Probability density functions (pdf) were calculated from the model and are illustrated superimposed onto the durations histogram to visually evaluate the fit. 

For macroscopic currents, peak (I_pk_) and steady state (I_ss_) current levels, as well as decay time constants for excised patches were determined using pClamp software based on the averages of (at least 5) traces obtained for each cell. Results were expressed as means ± SEM.

### Simulations

Macroscopic current traces were simulated as the time-dependent accumulation of receptors in open states. Initially, all receptors (200, 10 pA each) occupied the resting state and during the 1 mM glutamate pulse they bound glutamate with the rate constants determined previously for N1/N2A receptors [24]. Traces were analyzed similarly to the recorded macroscopic currents.

## Results

**Figure 1:**
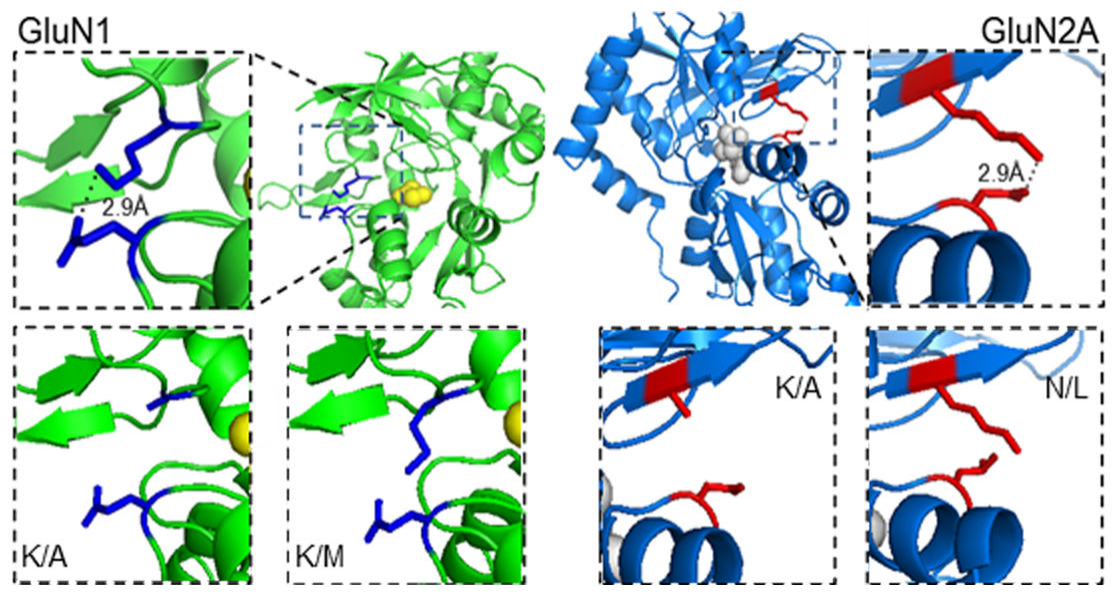
Putative cleft-spanning interactions in the LBD of GluN subunits. *Top*, proposed arrangements of the LBDs of glycine-bound GluN1(left, PDB 1PB7) and glutamate-bound GluN2A (*right*, PDB 2A5S) illustrate the position and cross-cleft distance of K483 and E712 (2.9 Å), and K487 and N687 side chains (2.9 Å), respectively. *Bottom*, PyMol representations of the substitutions examined in this study showing cross-cleft distances: *right*, N1^K/A^ 7.0 Å and N1^K/M^ 3.1 Å; *left*, N2^K/A^ 5.5 Å and N2^N/L^ 2.6 Å.

Crystal structures solved for the agonist-bound LBDs of N1 (PDB: 1PB7) and N2A (PBD: 2A5S) subunits illustrate clefts of similar but not identical geometries. In both structures, the ligands straddle the cleft and contact directly residues on both D1 and D2 lobes. In addition, the side chains of K483 and E712 in N1 and K487 and N687 in N2A extend across the cleft within 2.9 Å of one other, a distance consistent with non-covalent interactions [13] (Figure **1**). In AMPA receptors, functional measurements indicated that the homologous residue pairs interact across the cleft in full-length receptors and that these contacts influence the glutamate dissociation rate and the time course of AMPA receptor deactivation [21]. Similar studies in NMDA receptors showed that substitutions in the cleft alter the receptor’s apparent affinity for glutamate, but it remains unclear whether these residues contact each other in full-length receptors, and whether they affect the shape of the NMDA receptor response [25–27]. To investigate how these putative cross-cleft interactions contribute to NMDA receptor response kinetics, we perturbed the side-chains involved using single-residue mutagenesis and then examined the gating kinetics of the resulting receptors in the presence of supra-saturating concentrations of agonists. 

Using the published crystal structures as guides, we substituted alanine for lysine in the D1 of each subunit to produce N1(K483A) and N2A(K487A), herein referred to as N1^K/A^ and N2A^K/A^, respectively (Figure **1**). These mutations truncated the side chain of the D1 lysine residue in each putative pair and when examined in the static LBD structures produced cross-cleft distances of 7 Å and 5.5 Å, respectively, which are longer than required for cross-cleft interactions. Next, we used cell-attached patch-clamp recordings to investigate the activity patterns of full-length receptors containing these modified subunits. One-channel currents were recorded with the cell-attached patch-clamp technique in the presence of glutamate (1 mM) and glycine (0.1 mM), which ensured that regardless of possible effects on microscopic binding and dissociation rate constants, agonist re-binding would occur faster than the resolution limit of our analyses (0.15 ms), and, thus, our recordings will inform at all times about fully-liganded receptor states. 

**Figure 2:**
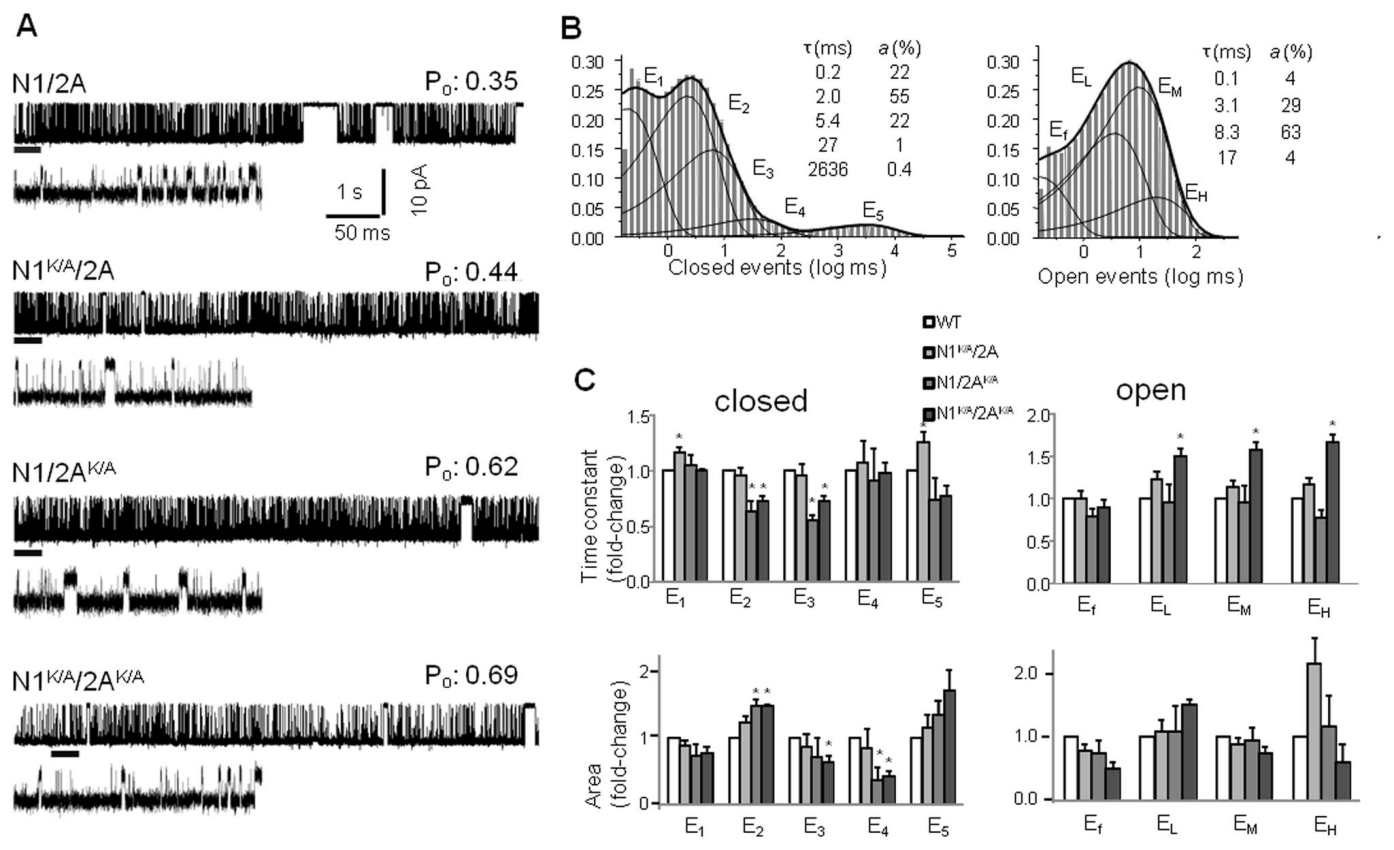
Single channel activity of NMDA receptors with side-chain truncation in the LBD cleft. (**A**) Currents were recorded from attached patches of HEK293 cells expressing N1^KE^/N2A^KN^, N1K/A/2A, N1/2A^K/A^, or N1K/A/2A^K/A^ receptors (**B**) Dwell time histograms for closed and open events recorded from one N1/N2A channel (799,601 events). Thin lines represent exponential components; insets give the time constants (τ) and relative areas (*a*) for these components. (C) Summary of changes in time constants (top) and areas (bottom) for closed and open components for the mutations examined. *, indicates significant changes relative to N1/2A receptors (p<0.05, Student’s *t*-test).

We obtained several long one-channel recordings (5 - 137 min) from N1^K/A^/N2A (n = 6), N1/N2A^K/A^ (n = 6), and N1^K/A^/N2A^K/A^ (n = 6) receptors expressed in HEK293 cells, and we compared these with traces recorded from wild-type (WT, N1/N2A) receptors (n = 13) (Figure **2**). Consistent with the relatively subtle changes we introduced in the receptors’ structure, we found that mutated receptors maintained WT-like distributions of the closed and open intervals recorded, which was indicative of an overall native activation mechanism consisting of five closed and four open states [28,29]. The traces in Figure 2 were selected for display because they have overall kinetic parameters (number of components and time constants) similar to the average behavior of each mutant receptor; however, given the limited duration, they represent necessarily only a snapshot of the complex receptor behaviors observed.

We were surprised to find that receptors carrying alanine substitutions, although presumably lacking stabilizing cross-cleft interactions, produced currents with *higher* open probabilities (P_o_) relative to WT receptors (Table **1**). When desensitized intervals (Figure **2B**: E_4_ and E_5_, which were not changed in duration) were excluded from analyses, the remaining bursts revealed more clearly slightly but significantly higher activities: by 8% for N1^K/A^/N2A and N1/N2A^K/A^, and by 14% for N1^K/A^/N2A^K/A^ (*p* < 0.04, Student’s t-test).

**Table 1:** Single channel parameters of NMDA receptors with substitutions within LBD clefts.

Receptor	distance (Å)		entire record				clusters			n
	N1	N2A	P_o_	MOT (ms)	MCT (ms)	events x10^6^	P_o_	MCT (ms)	events x10^6^	
N1/N2A	2.9	2.9	0.51 ± 0.03	6.7 ± 0.6	6.5 ± 0.8	4.1	0.74 ± 0.01	2.2 ± 0.1	4.1	13
N1K/A/2A	7	2.9	0.53 ± 0.07	9.2 ± 1.0*	8.5 ± 1.8	0.8	0.80 ± 0.03*	2.1 ± 0.2	0.8	6
N1/2A^K/A^	2.9	5.5	0.55 ± 0.05	6.5 ± 1.1	4.9 ± 0.4	1.6	0.80 ± 0.03*	1.3 ± 0.1*	1.6	6
N1K/A/2A^K/A^	7	5.5	0.59 ± 0.06	9.4 ± 0.8*	7.4 ± 2.0	2.2	0.84 ± 0.02*	1.6 ± 0.1*	2.3	6
N1^K/M^/2A	3.1	2.9	0.41 ± 0.06	6.1 ± 0.7	10.9 ± 2.4	3.1	0.69 ± 0.04	2.6 ± 0.3	3.1	10
N1/2A^N/L^	2.9	2.6	0.34 ± 0.06*	6.4 ± 0.8	15.6 ± 3.4*	1.9	0.63 ± 0.06*	3.6 ± 0.4*	1.9	9
**^*a*^**N1^CC^/2A	2.1	2.9	0.57 ± 0.05	6.5 ± 0.6	5.2 ± 0.7	3.7	0.72 ± 0.03			11
**^*a*^**N1/2A^CC^	2.9	2.1	0.69 ± 0.04*	7.6 ± 0.8	3.4 ± 0.7*	4.1	0.85 ± 0.02*			8
**^*a*^**N1^CC^/N2^CC^	2.1	2.1	0.64 ± 0.06	6.9 ± 1.5	6.3 ± 1.1	2.3	0.85 ± 0.02*			6

* indicates significant differences relative to N1/2A receptors (p<0.05, Student’s *t*-test).

^a^ reproduced from Kussius et al., 2010.

### Cross-cleft interactions in N1 and N2A LBDs

Inspecting the structures of open and closed interval distributions, we observed that although of similar magnitude, the K to A substitutions produced distinct and additive effects on NMDA receptor activity when introduced in N1 and/or N2A subunits. We traced the increased activity of N1^K/A^/N2A receptors solely to a 37% increase in mean open time (MOT, 9.2 ± 1.0 ms *vs.* WT, 6.7 ± 0.6 ms, p < 0.04), whereas that of N1/N2A^K/A^ receptors originated solely from a 41% decrease in intra-burst mean closed time (MCT, 1.3 ± 0.1 ms *vs.* WT, 2.2 ± 0.1 ms, p < 0.001). These strikingly specific subunit-dependent effects carried over in the double mutant, which produced activity with both longer MOT (9.4 ± 0.8 ms) and shorter intra-burst MCT (1.6 ± 0.1 ms). These values were indistinguishable from the N1^K/A^/N2A and N1/N2A^K/A^, respectively (p > 0.16), but were significantly different from WT-values (p < 0.02). 

Previously, we examined receptors with lobes that were covalently immobilized with disulfide bridges at cross-cleft distances comparable to the bonds illustrated by the LBD crystal structures and found that fixing the N1 lobes had no effect on channel kinetics whereas fixing the N2A lobes increased gating [30]. Based on these results, we surmised that in full-length receptors N1 LBDs organize in a manner that is similar to that illustrated by the crystal structure and, thus, further strengthening the cross-cleft interaction did not affect channel kinetics. However, in this study, we found that truncating the lysine side-chain and, thus, preventing the interaction between lysine and glutamate side-chains and increasing the cross-cleft distance increased channel activity by lengthening open durations. 

Existing views of glutamate receptor activation mechanism correlate an increase in agonist-elicited activity with an increase in the stability of closed-cleft conformations and/or to narrower cleft between D1 and D2 residues. The K-to-A substitutions we examined presumably eliminated the putative K-to-N interaction within the N2 pocket and simultaneously, according to the static crystal structure, created more cross-cleft space between these residues. Thus, the increased activity we observed may reflect not only the absence of interactions, but also the ability of the engineered cleft to become narrower than in wild-type receptors due to a reduction of steric hindrance. To investigate this possibility we designed and produced NMDA receptor subunits in which one of the residues within the native pair, K483/E712 in N1 and K487/N687 in N2A, were replaced with an isosteric residue that lacked charge.

### Isosteric substitutions within the LBD cleft

In N1, we replaced K483 on D1 with methionine to produce N1^K/M^, and in N2A we replaced N687 on D2 with leucine to produce N2A^N/L^ (Figure **1**). In the static arrangements depicted by the respective LBD structural models, these replacements maintained the close proximity of native residues within each pair: 3.1 Å between M483 and E742 in N1, and 2.6 Å between K487 and L687 in N2A, but were unable to connect the lobes through electrostatic forces. We reasoned that these more limited perturbations will help discriminate the effects of cross-cleft distance and bonding in NMDA receptor gating.

**Figure 3:**
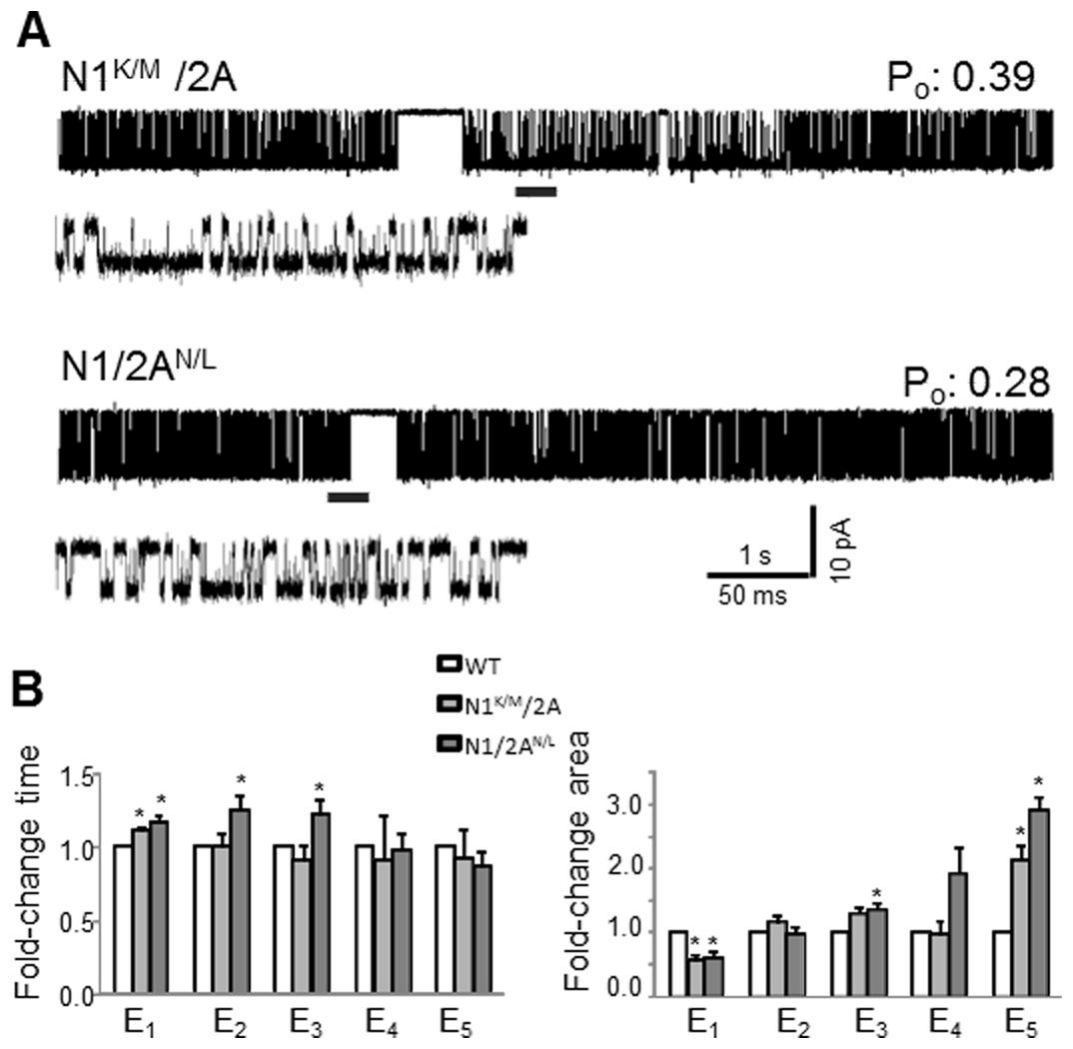
Single-channel currents of NMDA receptors with isosteric substitutions in the LBD pocket (A) Currents were recorded from attached patches on HEK293 cells expressing N1^K/M^/2A or N1/2A^N/L^ receptors. (**B**) Summary of changes in closed time constants (*left*) and areas (*right*). *, indicates significant changes relative to N1/2A receptors (p<0.05, Student’s *t*-test).

We expressed these mutated receptors in HEK293 cells and recorded currents from single receptors within a cell-attached membrane patch: N1^K/M^/N2A (n = 10, 3.1x10^6^ events) and N1/N2A^N/L^ (n = 9, 1.9x10^6^ events) (Figure **3A** and Table **1**). For all records considered, the distribution of the closed and open event durations resembled that of wild-type receptors, with five closed and four open states, demonstrating that the mutations did not alter the overall gating mechanism. 

Relative to wild-type, we found no significant differences in the P_o_ of N1^K/M^/N2A receptors, either across entire records or within bursts (0.4 ± 0.2 *vs.* WT, 0.5 ± 0.13 and 0.7 ± 0.1 vs. WT, 0.7 ± 0.1, respectively) (Table **1**). Based on this new result, we conclude that the putative cross-cleft *interaction* between the native lysine and aspartate residues of the N1 subunit does not contribute to the gating efficacy of NMDA receptors.

In contrast, we observed a significant decrease in the P_o_ for N1/N2A^N/L^ receptors. No change occurred within the open durations (data not shown) and the lower P_o_ resulted entirely from changes in the closed components similar to N1/N2^K/A^ receptors (Figure **3B** and Table **1**). This result supports the view that in wild-type receptors, lysine K487 and asparagine N687 of N2 subunits interact across the LBD cleft, and that this interaction likely stabilizes the cleft in the arrangement illustrated by the published crystal structure and contributes to the overall gating kinetics by causing closed durations to become shorter. Taken together, the results from receptors with isosteric mutations show that direct cross-cleft contacts in N1 and N2A LBDs contribute differentially to NMDA receptor gating [30–32]. 

### Role of cleft contacts in NMDA receptor gating

**Figure 4:**
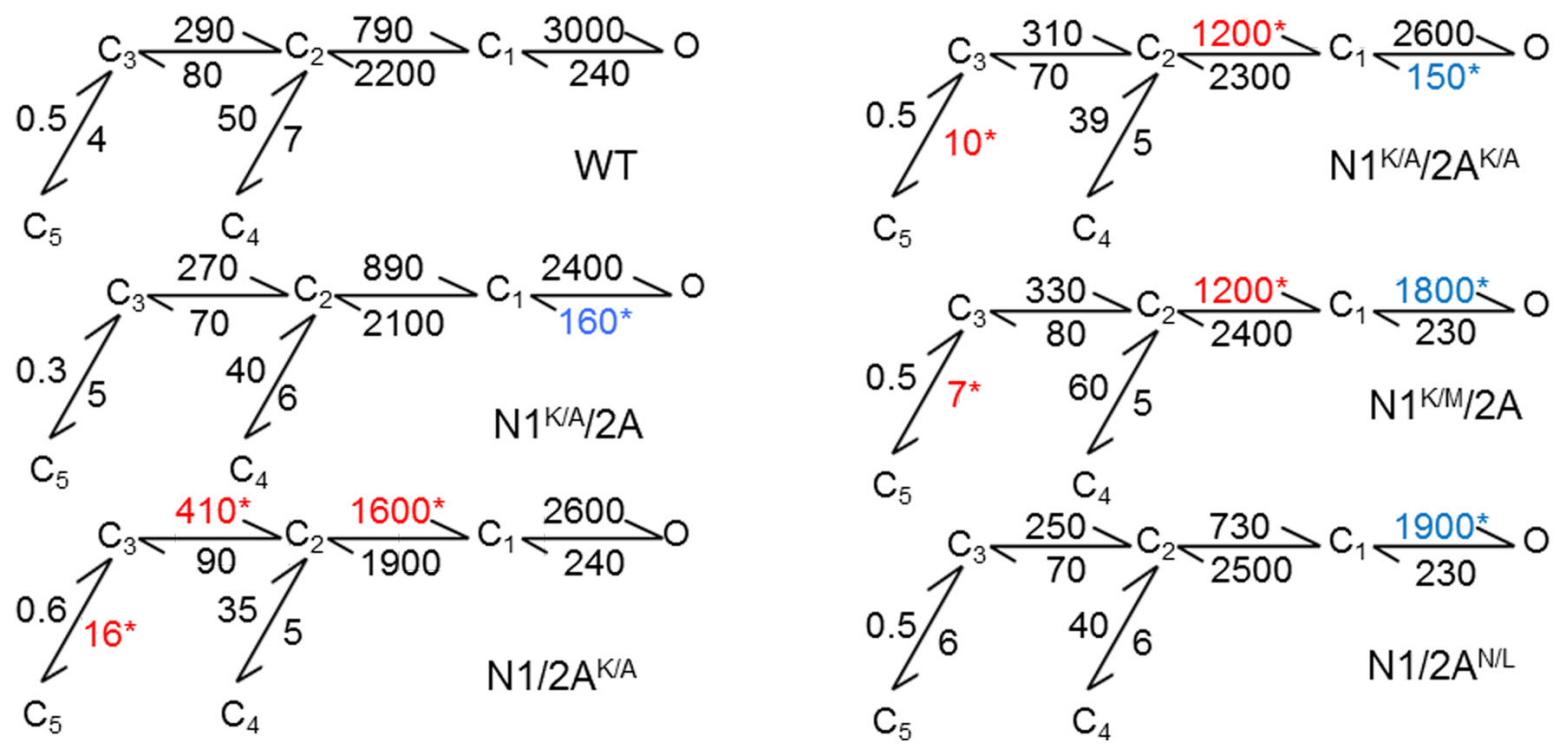
NMDA receptor gating reactions (A) Rate constants (s^-1^) were optimized with the illustrated 5C1O kinetic scheme from fits to single-channel data and are given as the rounded average for each data set. *, indicates significant changes (red, faster; blue, slower) relative to N1/2A receptors (p<0.05, Student’s *t*-test).

To quantify the respective contributions of LBD cross-cleft contacts on the NMDA receptor gating reaction, we used the single-channel data to fit kinetic model that includes five (pre-open and desensitized) closed states and one aggregated open state (Figure **4**) [33]. 

In N1^K/A^/N2A receptors, the only rate constant affected was that for the closing transition C_1_O, which was significantly slower (160 ± 20 s^-1^) compared to WT (240 ± 30 s^-1^, p < 0.05) (Figure **4**), reflecting the observed lengthening of open durations. In N1/N2A^K/A^ receptors, three transitions were altered: C_3_C_2_, C_2_C_1,_ and C_3_C_5_, in a manner consistent with the observed decrease in intra-burst closed times. In the double mutant N1^K/A^/N2A^K/A^ receptor, we observed the combined alterations noted in the single mutants, an indication that the effects of substitutions in N1 and N2A were additive.

Since there were no gross kinetic changes in the gating reaction of N1^K/M^/N2A receptors, we did not expect dramatic changes in rate constants. Several rates were significantly changed relative to WT, but these were in opposing directions, and, thus, had a nearly negligible effect on the overall gating characteristics of these mutants. The slower activity of N1/N2A^N/L^ receptors was traced to the slowing of a single transition, C_1_ O, from 3,000 ± 300 s^-1^ to 1,900 ± 200 s^-1^ (Figure **4**). Interestingly, this transition was unaffected in N1/N2A^K/A^ receptors. This observation is consistent with a scenario where the native interaction between lysine and asparagine residues contributes significantly to the stability of a closed N2A cleft conformation, and, in turn, a closed-cleft conformation is favorable for efficient gating. In contrast, in the K/A mutants, although the K-N interaction is also absent, a shorter side chain promotes enhanced gating presumably by allowing further cleft closure (Table **1**) [30]. In this scenario, a narrow N2A cleft, as in the N1/N2A^K/A^ mutant, may accelerate activation (faster *k*_C3C2_ and *k*_C2C1_), whereas a less stable closed cleft, as in the N1/N2A^N/L^ mutant, slows activation (slower *k*_C1O_).

**Figure 5:**
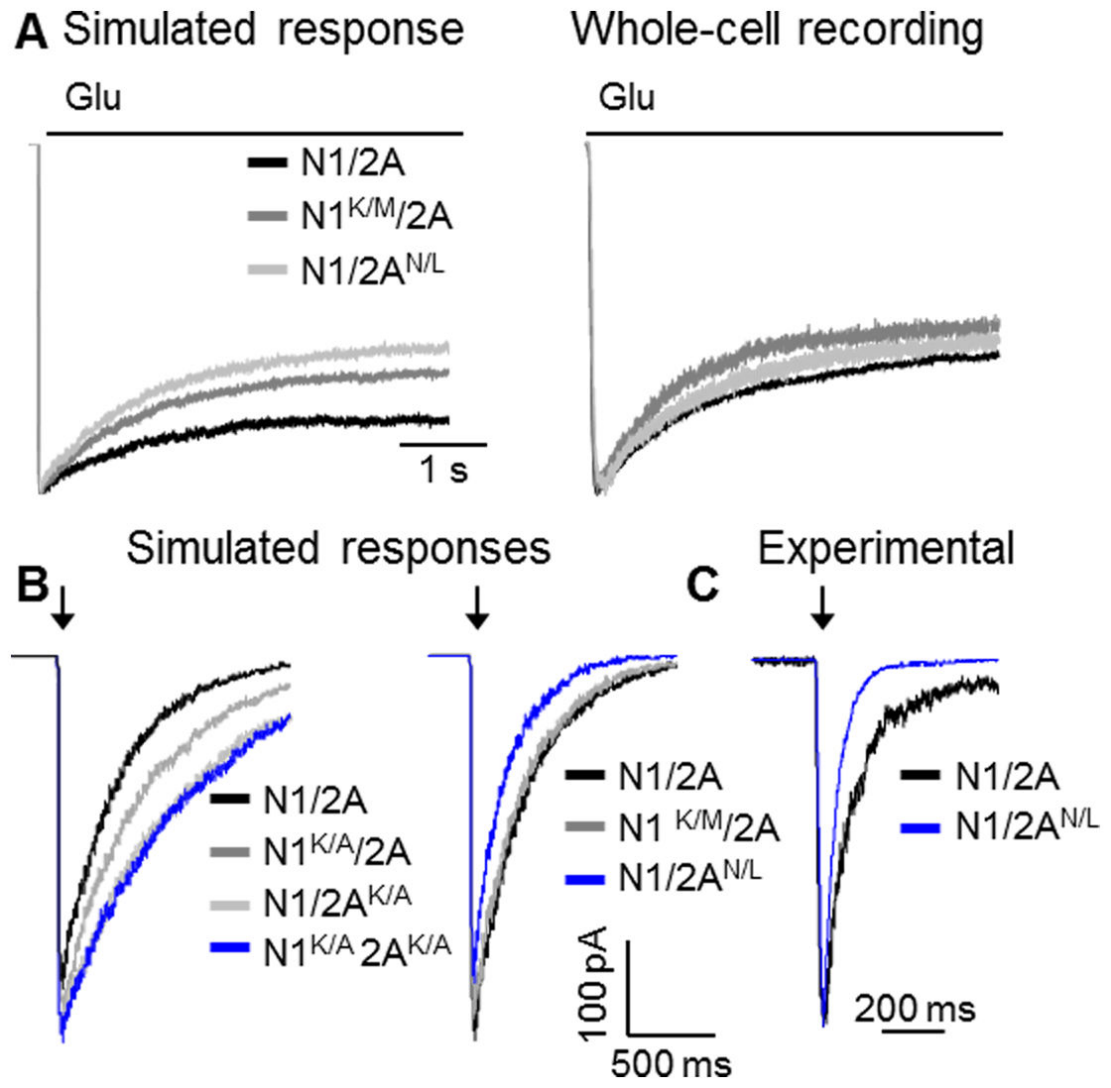
Macroscopic NMDA receptor currents. (A) Responses to long pulses of glutamate (5 s, 1 mM) were simulated with the models in Figure 4 (*left*) or were recorded as whole-cell currents from HEK293 cells expressing the indicated receptors (*right*). (**B**) Synaptic-like responses were simulated with the models in Figure 4. (**C**) Macroscopic responses were recorded from outside-out patches pulled from HEK293 cells expressing WT or N1/2A^N/L^ receptors in response to brief exposure to glutamate (arrow: 10 ms, 1 mM); experimental traces were normalized to peak and overlaid for comparison.

If, as our single-channel observations indicate, the LBD cross-cleft interactions contribute to gating, it is important to determine how these structural features influence the NMDA receptor macroscopic response. A direct evaluation of how the mutations affect the amplitude of the ensemble current is not feasible experimentally because when measuring whole-cell currents the total number of receptors varies from one cell to the next and is unknown. Instead, macroscopic measurements inform about possible changes in the time course of the ensemble response. In an attempt to test our models, we simulated macroscopic currents with long (5 s) pulses of glutamate (1 mM), and we found largely WT-like kinetics for the K/A mutants, N1^K/A^/N2A and N1/N2A^K/A^, and a small increase in macroscopic desensitization for the N1^K/M^/N2A and N1/N2A^N/L^ receptors (Figure **5A**). To test this prediction, we recorded whole-cell currents elicited by long (5 s) glutamate application (1 mM) and given the difference in experimental conditions (cell-attached for simulations *vs.* whole-cell for experiments) we observed a satisfactory concordance between model predictions and experimental measurement. 

Next, we used the kinetic models we developed to evaluate how the LBD cross-cleft interactions may impact responses to brief (10 ms) synaptic-like exposures to glutamate (Figure **5B**). Relative to WT, both K/A substitutions resulted in currents with larger peaks, whereas the isosteric mutations, N1^K/M^/N2A and N1/N2A^N/L^, produced currents with similar or lower peak levels, respectively. More significantly, the deactivation rates were slower for both K/A substituted receptors, whereas the deactivation rate was increased for N1/N2A^N/L^. We should note that for these simulations we assumed that the microscopic association and dissociation rates for glutamate were not affected by the mutations, and, thus, the results only reflect the changes in gating we estimated in this study. To compare these simulated traces with macroscopically recorded responses, we measured currents from N1/N2A^N/L^ receptors (n=4) residing in outside-out patches. Small membrane patches were pulled and exposed to 10 ms pulses of 1mM glutamate in the presence of continuous 0.1 mM glycine (Figure **5C**). Consistent with our simulation results, N1/N2A^N/L^ had faster deactivation time-constants relative WT receptors observed in the same conditions (280 ± 40 ms *vs.* 120 ± 13 ms), respectively [34]. Based on these results, we surmise that in NMDA receptors cross-cleft contacts, whether steric or electrostatic, control the peak level and the deactivation time course of the current, regardless of possible additional effects on agonist affinity. 

## Discussion

In this study, we investigated the role of putative direct D1-D2 interactions within the LBDs of NMDA receptors and determined that the side chains examined contributed substantially to setting the time course of receptor deactivation. When a single lysine residue was replaced with alanine in either N1 (K483A) or N2A (K487A), presumably abolishing a native cross-cleft interaction and reducing bulk at the cross-cleft interface, gating efficiency was enhanced resulting in macroscopic currents with larger peaks and slower deactivation. In contrast, when the interaction was disrupted by eliminating the side chain charge on in N1^K/M^ or N2A^N/L^, gating efficiency was unaffected if the mutation was in N1 and decreased, when the mutation was in N2A, the latter resulting in macroscopic currents with smaller peaks and faster deactivation. These results reinforce the distinct roles in NMDA receptor gating played by N1 or N2A cleft residues.

Previous studies have suggested that the stability of a narrow LBD cleft determines, in part, agonist efficacy [35]. Thus, we were surprised to find that NMDA receptor activity was increased in the K/A mutants, as this substitution was predicted to eliminate a stabilizing cross-cleft non-covalent bond. A possible explanation is that minimizing steric bulk at this location within the interface may allow the LBD to close further or to increase cleft stability by permitting alternate interactions or changing the hydrophobic environment within the cleft. This interpretation is consistent with the present view that additional closure correlates with increased activity, a concept originating from measurements of lobe rotation in crystal structures of isolated GluA2 LBDs in complex with partial agonists, full agonists, and antagonists [10,20]. For NMDA receptors, however, the validity of the ‘more closure more activity’ model was challenged by similar measurements in isolated N1-LBDs where glycine, a full agonist, or D-cycloserine, a partial agonist, produced the same degree of cleft-closure [11]. Notably, the same direct D1-D2 interactions were predicted to form in the partial agonist and glycine-bound N1 LBDs [13]. Based on the data presented here that disrupting the electrostatic interaction between K483 and E712 in N1 does not contribute significantly to the efficacy of NMDA receptor gating, we propose that this salt bridge, rather than preventing the lobes from moving apart, acts as a ‘spacer’ that prevents them from collapsing further, and provides the tension necessary for channels to deactivate. This view is supported by two main results. First, when we replaced K483 with methionine, a substitution that preserves the D1-D2 distance but precludes electrostatic interactions, we found no significant change in NMDA receptor activation. This result may indicate that the predicted electrostatic interaction between the K483/E712 does not occur in full length receptors. Second, when we locked the N1 LBDs with disulfide bridges at the distances suggested by the structural model of soluble agonist-LBD complexes, the resulting receptors, denoted as N1^CC^/N2, were constitutively active and had WT-like kinetics (Table **1**, P_o_: 0.57 ± 0.05 *vs.* 0.51 ± 0.03) [30], an indication that native receptors have arrangements similar to those illustrated by the glycine-bound N1-LBD crystal structure. Thus, as long as the D1-D2 distance was preserved, eliminating or strengthening the N1 D1-D2 interaction had little consequence on NMDA receptor gating. A corollary of these results is that in full-length receptors glycine stabilizes N1-LBD conformations that are similar to those observed in crystals of detached LBDs. 

In contrast, our results are consistent with the view that the D1-D2 interaction illustrated in the N2A LBD crystal structure between K487 and N687 contributes substantially to the shape of the glutamate elicited response of NMDA receptors. Exchanging N687 for leucine, which eliminates a hydrogen bond and adds bulk, significantly impaired NMDA receptor gating. However, a covalent bond engineered to fix these residues at the distance illustrated by the N2A LBD crystal structure, referred to as N1/N2^CC^, produced significantly increased gating (Table **1**, P_o_: 0.69 ± 0.04 *vs.* 0.51± 0.03). Further, the side chain truncation at K487 and the N1/N2^CC^ increased gating efficiency with the same mechanism: a significant decrease in MCT (Table **1**, 3.4 ± 0.7 ms *vs.* 6.5 ± 0.8 ms). In addition, exchanging N687 for aspartate, which contributes a negative charge and promotes electrostatic contact, gating was also enhanced [25,27,30]. These results suggest that in full-length receptors glutamate facilitates conformations of the LBDs that are more open or less stable than those depicted by the crystal structure of the soluble glutamate-LBD complex. Importantly, our results from the alanine and the isosteric mutants suggest that residues at the D1-D2 interface finely tune the shape of the NMDA receptor response to agonist. This interaction can define both the extent to which the LBD can close and its stability once it is closed. Both are important factors in regulating the peak and time-course of the NMDA receptor response.

An important observation in this study was that cross-cleft interactions we studied did not alter NMDA receptor modal behavior, as measured by the relative abundance of the three types of openings. In AMPA receptors modal behavior varies with agonist concentrations and is affected by mutations within the LBD cleft [36]. Our results add to the already existing evidence that modal behavior arises by separate mechanisms in AMPA and NMDA receptors.

Lastly, a consistent finding across several studies is that similar perturbations in N1 and N2A subunits make distinct contributions to gating. In the current study, we found the alterations in N1 and N2 to have additive effects suggesting independent contributions to gating by the N1 and N2 LBDs. Although agonist-binding to both subunits results in cleft closure each subunit implements its closed-cleft conformation via distinct interactions, these have separate contributions to the gating reaction, with N1 interactions in affecting the stability of the open states and N2 affecting mostly closed durations.

In summary, our results demonstrate that changing the size or charge of residues facing the LBD clefts of NMDA receptors that may affect closed-cleft dimensions and/or stability have distinct and measurable effects on receptor gating. Thus, the interactions between mobile lobes are important determinants of gating efficacy, and they contribute to the characteristically slow deactivation kinetics of NMDA receptors. 

## References

[1] Cull-Candy S, Brickley S, Farrant M (2001) NMDA receptor subunits: diversity, development and disease. Curr Opin Neurobiol 11: 327-335. doi:10.1016/S0959-4388(00)00215-4. PubMed: 11399431. 11399431

[2] Lester RA, Clements JD, Westbrook GL, Jahr CE (1990) Channel kinetics determine the time course of NMDA receptor-mediated synaptic currents. Nature 346: 565-567. doi:10.1038/346565a0. PubMed: 1974037. 1974037

[3] Jahr CE (1992) High probability opening of NMDA receptor channels by L-glutamate. Science 255: 470-472. doi:10.1126/science.1346477. PubMed: 1346477. 1346477

[4] Popescu G, Robert A, Howe JR, Auerbach A (2004) Reaction mechanism determines NMDA receptor response to repetitive stimulation. Nature 430: 790-793. doi:10.1038/nature02775. PubMed: 15306812. 15306812

[5] Traynelis SF, Wollmuth LP, McBain CJ, Menniti FS, Vance KM et al. (2010) Glutamate Receptor Ion Channels:Structure, Regulation, and Function. Pharmacol Rev 62: 405-496. doi:10.1124/pr.109.002451. PubMed: 20716669. 20716669 PMC2964903

[6] Laube B, Kuhse J, Betz H (1998) Evidence for a tetrameric structure of recombinant NMDA receptors. J Neurosci 18: 2954-2961. PubMed: 9526012. 9526012 10.1523/JNEUROSCI.18-08-02954.1998 PMC6792599

[7] Rosenmund C, Stern-Bach Y, Stevens CF (1998) The tetrameric structure of a glutamate receptor channel. Science 280: 1596-1599. doi:10.1126/science.280.5369.1596. PubMed: 9616121. 9616121

[8] Abele R, Keinanen K, Madden DR (2000) Agonist-induced isomerization in a glutamate receptor ligand-binding domain. A kinetic and mutagenetic analysis. J Biol Chem 275: 21355-21363. doi:10.1074/jbc.M909883199. PubMed: 10748170. 10748170

[9] Cheng Q, Du M, Ramanoudjame G, Jayaraman V (2005) Evolution of glutamate interactions during binding to a glutamate receptor. Nat Chem Biol 1: 329-332. doi:10.1038/nchembio738. PubMed: 16408071. 16408071

[10] Armstrong N, Sun Y, Chen GQ, Gouaux E (1998) Structure of a glutamate-receptor ligand-binding core in complex with kainate. Nature 395: 913-917. doi:10.1038/27692. PubMed: 9804426. 9804426

[11] Furukawa H, Gouaux E (2003) Mechanisms of activation, inhibition and specificity: crystal structures of the NMDA receptor NR1 ligand-binding core. EMBO J 22: 2873-2885. doi:10.1093/emboj/cdg303. PubMed: 12805203. 12805203 PMC162155

[12] Furukawa H, Singh SK, Mancusso R, Gouaux E (2005) Subunit arrangement and function in NMDA receptors. Nature 438: 185-192. doi:10.1038/nature04089. PubMed: 16281028. 16281028

[13] Inanobe A, Furukawa H, Gouaux E (2005) Mechanism of partial agonist action at the NR1 subunit of NMDA receptors. Neuron 47: 71-84. doi:10.1016/j.neuron.2005.05.022. PubMed: 15996549. 15996549

[14] Kuusinen A, Arvola M, Keinänen K (1995) Molecular dissection of the agonist binding site of an AMPA receptor. EMBO J 14: 6327-6332. PubMed: 8557052. 8557052 10.1002/j.1460-2075.1995.tb00323.x PMC394757

[15] Mayer ML (2006) Glutamate receptors at atomic resolution. Nature 440: 456-462. doi:10.1038/nature04709. PubMed: 16554805. 16554805

[16] Lau AY, Roux B (2011) The hidden energetics of ligand binding and activation in a glutamate receptor. Nat Struct Mol Biol 18: 283-287. doi:10.1038/nsmb.2010. PubMed: 21317895. 21317895 PMC3075596

[17] Speranskiy K, Kurnikova M (2005) On the binding determinants of the glutamate agonist with the glutamate receptor ligand binding domain. Biochemistry 44: 11508-11517. doi:10.1021/bi050547w. PubMed: 16114887. 16114887

[18] Jin R, Horning M, Mayer ML, Gouaux E (2002) Mechanism of activation and selectivity in a ligand-gated ion channel: structural and functional studies of GluR2 and quisqualate. Biochemistry 41: 15635-15643. doi:10.1021/bi020583k. PubMed: 12501192. 12501192

[19] Gill A, Birdsey-Benson A, Jones BL, Henderson LP, Madden DR (2008) Correlating AMPA receptor activation and cleft closure across subunits: crystal structures of the GluR4 ligand-binding domain in complex with full and partial agonists. Biochemistry 47: 13831-13841. doi:10.1021/bi8013196. PubMed: 19102704. 19102704 PMC2629381

[20] Armstrong N, Gouaux E (2000) Mechanisms for activation and antagonism of an AMPA-sensitive glutamate receptor: crystal structures of the GluR2 ligand binding core. Neuron 28: 165-181. doi:10.1016/S0896-6273(00)00094-5. PubMed: 11086992. 11086992

[21] Robert A, Armstrong N, Gouaux JE, Howe JR (2005) AMPA receptor binding cleft mutations that alter affinity, efficacy, and recovery from desensitization. J Neurosci 25: 3752-3762. doi:10.1523/JNEUROSCI.0188-05.2005. PubMed: 15829627. 15829627 PMC6724928

[22] Kussius CL, Popescu GK (2009) Kinetic basis of partial agonism at NMDA receptors. Nat Neurosci 12: 1114-1120. doi:10.1038/nn.2361. PubMed: 19648915. 19648915 PMC2739723

[23] Qin F (2004) Restoration of single-channel currents using the segmental k-means method based on hidden Markov modeling. Biophys J 86: 1488-1501. doi:10.1016/S0006-3495(04)74217-4. PubMed: 14990476. 14990476 PMC1303984

[24] Popescu G, Robert A, Howe JR, Auerbach A (2004) Reaction mechanism determines NMDA receptor response to repetitive stimulation. Nature 430: 790-793. doi:10.1038/nature02775. PubMed: 15306812. 15306812

[25] Maier W, Schemm R, Grewer C, Laube B (2007) Disruption of interdomain interactions in the glutamate binding pocket affects differentially agonist affinity and efficacy of N-methyl-D-aspartate receptor activation. J Biol Chem 282: 1863-1872. PubMed: 17105731. 17105731 10.1074/jbc.M608156200

[26] Kalbaugh TL, VanDongen HM, VanDongen AM (2004) Ligand-binding residues integrate affinity and efficacy in the NMDA receptor. Mol Pharmacol 66: 209-219. doi:10.1124/mol.66.2.209. PubMed: 15266011. 15266011

[27] Blanke ML, VanDongen AM (2008) Constitutive activation of the N-methyl-D-aspartate receptor via cleft-spanning disulfide bonds. J Biol Chem 283: 21519-21529. doi:10.1074/jbc.M709190200. PubMed: 18450751. 18450751 PMC2490796

[28] Stern P, Cik M, Colquhoun D, Stephenson FA (1994) Single channel properties of cloned NMDA receptors in a human cell line: comparison with results from Xenopus oocytes. J Physiol (Lond) 476: 391-397. 8057248 10.1113/jphysiol.1994.sp020140 PMC1160453

[29] Popescu G, Auerbach A (2003) Modal gating of NMDA receptors and the shape of their synaptic response. Nat Neurosci 6: 476-483. PubMed: 12679783. 12679783 10.1038/nn1044

[30] Kussius CL, Popescu GK (2010) NMDA receptors with locked glutamate-binding clefts open with high efficacy. J Neurosci 30: 12474-12479. doi:10.1523/JNEUROSCI.3337-10.2010. PubMed: 20844142. 20844142 PMC3423094

[31] Kussius CL, Popescu AM, Popescu GK (2010) Agonist-specific gating of NMDA receptors. Channels 4: 1-5. doi:10.4161/chan.4.1.11537. PubMed: 20368689. 19934647 PMC3150751

[32] Kussius CL, Popescu GK (2009) Kinetic basis of partial agonism at NMDA receptors. Nat Neurosci 12: 1114 - 1120. doi:10.1038/nn.2361. PubMed: 19648915. 19648915 PMC2739723

[33] Kussius CL, Kaur N, Popescu GK (2009) Pregnanolone sulfate promotes desensitization of activated NMDA receptors. J Neurosci 29: 6819-6827. doi:10.1523/JNEUROSCI.0281-09.2009. PubMed: 19474309. 19474309 PMC3150747

[34] Maki BA, Aman TK, Amico-Ruvio SA, Kussius CL, Popescu GK (2012) C-terminal Domains of N-Methyl-D-aspartic Acid Receptor Modulate Unitary Channel Conductance and Gating. J Biol Chem 287: 36071-36080. doi:10.1074/jbc.M112.390013. PubMed: 22948148. 22948148 PMC3476275

[35] Jin R, Banke TG, Mayer ML, Traynelis SF, Gouaux E (2003) Structural basis for partial agonist action at ionotropic glutamate receptors. Nat Neurosci 6: 803-810. doi:10.1038/nn1091. PubMed: 12872125. 12872125

[36] Popescu GK (2012) Modes of glutamate receptor gating. J Physiol 590: 73-91. PubMed: 22106181. 22106181 10.1113/jphysiol.2011.223750 PMC3300047

